# PLAID: ultrafast single-sample gene set enrichment scoring

**DOI:** 10.1093/bioinformatics/btaf621

**Published:** 2025-11-12

**Authors:** Antonino Zito, Xavier Escribà Montagut, Gabriela Scorici, Axel Martinelli, Murodzhon Akhmedov, Ivo Kwee

**Affiliations:** BigOmics Analytics, Via Serafino Balestra 12, Lugano, 6900, Switzerland; BigOmics Analytics, Via Serafino Balestra 12, Lugano, 6900, Switzerland; BigOmics Analytics, Via Serafino Balestra 12, Lugano, 6900, Switzerland; BigOmics Analytics, Via Serafino Balestra 12, Lugano, 6900, Switzerland; BigOmics Analytics, Via Serafino Balestra 12, Lugano, 6900, Switzerland; BigOmics Analytics, Via Serafino Balestra 12, Lugano, 6900, Switzerland

## Abstract

**Summary:**

In recent years, computational methods have emerged that calculate enrichment of gene signatures within individual samples. These signatures offer critical insights into the coordinated activity of functionally related genes, proteins or metabolites, enabling the identification of unique molecular profiles in individual cells and patients. This strategy is pivotal for patient stratification and advancement of personalized medicine. However, the rise of large-scale datasets, including single-cell profiles and population biobanks, has exposed significant computational inefficiencies in existing methods. Current methods often demand excessive runtime and memory resources, becoming impractical for large datasets. Overcoming these limitations is a focus of current efforts by bioinformatics teams in academia and the pharmaceutical industry, as essential to support basic and clinical biomedical research. To address this critical need, we developed PLAID (Pathway Level Average Intensity Detection), an ultrafast and memory optimized single sample gene set enrichment algorithm that utilizes sparse matrix computation. PLAID delivers highly accurate gene set scoring and surpasses the performance of current methods in single-cell and bulk transcriptomics, and proteomics data. PLAID uniquely integrates the most widely used gene set scoring algorithms, enabling researchers to apply multiple methods for cross-validation with outstanding runtime efficiency and minimal memory requirement.

**Availability and implementation:**

PLAID is implemented in the R language for statistical computing. PLAID source code and installation instructions are available with no restrictions at https://github.com/bigomics/plaid.

## 1 Introduction

Single sample analyses, including at single cell resolution, are advancing our understanding of biology (Haque *et al.* 2017, Van de Sande *et al.* 2023, Zheng *et al.* 2025). Single-cell transcriptomic and epigenomic profiles reveal disease-related molecular signatures and inter-individual differences, enabling personalized medicine approaches (Sheridan 2024, Pekayvaz *et al.* 2025). While single-gene studies have revealed individual dysregulated factors, complex diseases may involve heterogenous pathophysiology driven by multi-factorial perturbations in cellular networks. Because these networks regulate homeostasis, dysregulations within a gene set can impact critical cellular functions (Yang 2020, Galindez *et al.* 2023). Targeted therapies are likely to be more effective if they restore the function of the entire pathway, rather than single genes alone. Characterizing these pathways enables patient stratification and personalized treatments (Fröhlich *et al.* 2018).

Curated collections such as MSigDB (Liberzon *et al.* 2015) and Reactome (Milacic *et al.* 2024), serve as references for gene set scoring. Enrichment methods map genes to these collections and compute gene set enrichment scores (GSESs), whose statistical significance can be assessed using competitive or self-contained null hypothesis tests (Goeman and Bühlmann 2007). While earlier methods like GSEA (Subramanian *et al.* 2005), scored gene sets at population level, newer methods perform scoring in each sample or cell (Barbie *et al.* 2009, Hänzelmann *et al.* 2013, Aibar *et al.* 2017, Foroutan *et al.* 2018, Pont *et al.* 2019, Andreatta and Carmona 2021). Using the ranks of individual samples for gene set scoring avoids the need to recalculate GSESs when integrating new samples. Earlier approaches were mostly supervised, modeling GSESs on discrete or continuous traits. In contrast, unsupervised methods assess coordinately down- or up-regulated signatures based on relative ranks rather than absolute expression. Another method, GSVA (Hänzelman *et al.* 2013), leverages global profiles to evaluate low or high expression in each sample, normalizing to a Gaussian or discrete Poisson distribution.

**Figure 1.**
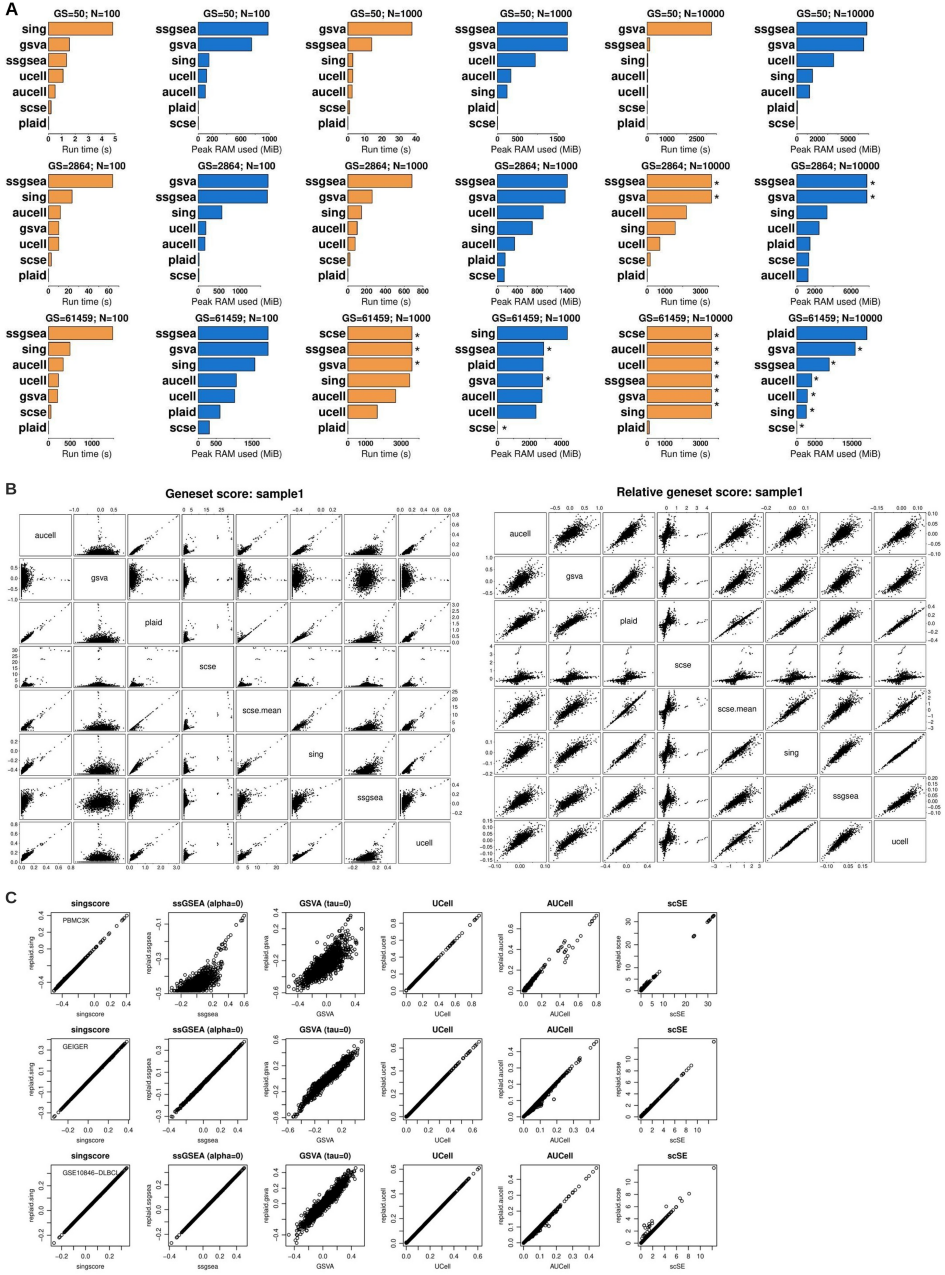
Benchmarking PLAID runtime, memory, and GSESs against singscore, ssGSEA, GSVA, Ucell, AUCell, and scSE. (A) Run time (s) and peak RAM memory (MiB) use [peakRAM R package (Quinn 2017)]. The PBMC scRNA-seq data (∼2700 cells; available from 10X Genomics and Seurat R package (Hao *et al.* 2024)] was used. Benchmarking involved increasing sizes: 100, 1K, 10K cells (this latter generated by replication up to 10K); 50 gene sets, corresponding to the “HALLMARK” Omics Playground (OPG) collection; 2864 gene sets, corresponding to the “GO_BP” OPG collection; 61 459 gene sets, corresponding to the whole OPG collection. Runs were timed out at 1 h. The *x* axis shows PLAID peak memory or runtime. The *y* axis shows the method. For timed out runs, the effective peak memory could not be correctly estimated. Timed out runs are indicated by an asterisk. (B) Scatter plots between GSESs calculated by PLAID versus singscore, ssGSEA, GSVA, UCell, AUCell, scSE, and scSE.mean. Original (left) and row-centered GSESs (right) are shown. 2864 GO_BP gene sets were used for analysis. The *x* and *y* axis report the GSES values. (C) Scatter plots between original and PLAID-replicated GSESs for singscore, ssGSEA, GSVA, UCell, AUCell, scSE. The *x* and *y* axis report the GSES values. Three datasets were used: (top to bottom): PBMC scRNA-seq; LC/MS proteomics (Wolf *et al.* 2020); mRNA microarray (GSE10846, Lenz *et al.* 2008). PLAID replicates distinct methods while being faster and more memory efficient.

A common drawback of most single-sample enrichment methods is their computational inefficiency as sample size grows. This issue is particularly pronounced with the rise of large-scale biological data. Both ssGSEA and GSVA are impractical in terms of runtime and memory usage. For instance, ssGSEA requires over 600 s to process 2864 gene sets across 1K single cells on a modern multi-core, 48GB RAM laptop (Fig. 1A). To address these limitations, more efficient approaches have been developed. Single-cell Signature Explorer (scSE) computes a score for each single cell transcriptome and a given gene set as the sum of all UMIs for the genes in the gene set, divided by the total UMI count in the cell. Relying on sum -which is computationally efficient- and developed in Go language, scSE computes 13 million scores (1K gene sets; 13K cells) in under 5 min, outperforming several approaches (Pont *et al.* 2019). Ucell takes a different approach, scoring gene signatures using the Matt-Whitney U test (Mann and Whitney 1947, Andreatta and Carmona 2021). Specifically, genes are ranked and a pseudo-rank of 1 added to mitigate zero-inflated counts. Despite these advances, the rapid growth of population-scale data, comprising hundreds of thousands of samples (cells), implies that computational inefficiency remains a major barrier. Development of scalable and memory efficient algorithms is essential to avoid analytical bottlenecks and to drive progresses in precision medicine and systems biology.

To address the pressing need for scalable and efficient GSES methods, we developed PLAID (Pathway Level Average Intensity Detection). PLAID scores a gene set based on the average intensity of its constituent genes within each sample. PLAID is an ultrafast and memory efficient solution due to optimized use of sparse matrices. PLAID delivers GSESs that are highly concordant with existing methods, while achieving up to 100-fold gain in computational efficiency. We conducted extensive testing of PLAID using single-cell and bulk transcriptomics, and proteomics data, and found it outperforms the widely used singscore (Foroutan *et al.* 2018), ssGSEA (Barbie *et al.* 2009), GSVA (Hänzelmann *et al.* 2013), UCell (Andreatta and Carmona 2021) and AUCell (Aibar *et al.* 2017). Performing validation of enriched gene sets through independent methods is critical for robust analysis. Unfortunately, for large datasets, this remains a challenging step with current methods. To provide a solution, we have implemented a unified framework including singscore, ssGSEA, GSVA, scSE, UCell, and AUCell within PLAID. This architecture enables validation with multiple methods while also providing much improved speed and memory efficiency.

PLAID not only correlates strongly with established methods but also offers unmatched computational efficiency, making it uniquely suited for large-scale applications. PLAID represents a significant advancement in calculating single-sample GSES by combining accuracy, scalability, and reproducibility with exceptional speed and resource efficiency.

## 2 Methods

### 2.1 PLAID algorithm

PLAID requires: (i) log-transformed data matrix (gene, protein, metabolite expression), with features on rows and samples on columns, herein referred to as *X*. Ideally, *X* is a sparse matrix; (ii) a sparse gene set matrix with features on rows and gene sets on columns, herein referred to as *G.* The use of sparse matrices is a key contribution to providing a computationally efficient GSES approach. The whole *G* matrix corresponds to the whole Omics Playground (OPG) collection of gene sets (Akhmedov *et al.* 2020), which derives from multiple established, widely used public collections. For instance, it includes *N* = 50 “Hallmarks,” *N* = 2864 “GO_BP,” *N* = 264 “GO_CC,” *N* = 503 “GO_MF,” *N* = 955 “REACTOME,” *N* = 373 “WikiPathways,” and *N* = 120 “MSigDb” gene sets. These collections remain available in the OPG with no restrictions. Often, gene sets are available as gmt (Gene Matrix Transposed), which can be converted into a sparse matrix using the provided *gmt2mat* function. *X* and *G* are filtered to include only shared features, as by PLAID. From the *G* matrix, a binary indicator matrix of non-zero elements is created, providing information on which gene is present in each gene set. Afterwards, the sum of each column in *G* is computed using the *Matrix::colSums* R function which handles sparse matrices. Missing values, if present, are excluded from calculation. A 1e-8 offset is added to prevent full zeros. Each column *of G* is then scaled by its sum using the *Matrix::colScale* R function to handle sparse matrices. The normalization of *G* ensures that each column of *G* sums to 1. PLAID single-sample GSESs are calculated using the matrix cross-product between *G* and *X*, as follows:


(1) $$\text{crossprod}\left(G,X\right)=G^{T}X\;\text{PLAID }\;\text{single}-\text{sample}\;\text{GSES}$$

Equation (1) is equivalent to the R call t(*G*) %*% *X*. Mathematically, the formula above is the core computation of PLAID, corresponding to the average log-intensity of all features within a gene set in a sample. For large number of gene sets (e.g. >10K) or cells (e.g. >100K), PLAID is equipped with functionalities to calculate the matrix cross-product in chunks to avoid memory burden. Large matrices are automatically detected by PLAID and chunk computation activated. Specifically, an initial chunk size is estimated based on machine integer limits (calculated using the built-in R variable *.Machine$integer.max)* and the number of columns of the *G* matrix. The number of samples in *X* is also checked: if it is equal or above the estimated chunk size, chunk computation is automatically activated. In chunk computation, the matrix cross-product to compute PLAID GSESs is performed per chunks of samples. Results from all chunks are combined into a unified matrix which is subject to normalization. The automatic chunk computation is a key contribution to providing a computationally efficient scoring method. PLAID does not center nor ranks the input matrix. It outputs the results as a score matrix (gene sets in rows; samples in columns).

### 2.2 Normalization of PLAID gene set enrichment scores

The output of PLAID is a matrix of GSESs. By default (when e.g >20 gene sets tested), PLAID applies median normalization of the results. Each column is median-centered. The overall mean of the column medians is then added. Because GSESs are normalized, it is not strictly necessary to normalize the input matrix *X*. The median-normalized gene set matrix can also be used for differential gene set expression testing between groups.

### 2.3 Sparse matrix computations

PLAID significantly relies on sparse matrix computation. It uses the Matrix R package (Bates *et al.* 2024), which can operate on sparse matrices, wrapping the “SuiteSparse” libraries. Single-cell data are preferably represented as sparse matrices. It is highly recommended to use sparse matrices as this takes advantage of the PLAID’s superior computational efficiency for gene set scoring. The functions *gmt2mat* and *mat2gmt* allow to convert a gmt list to a sparse matrix and vice versa. Creating the sparse matrix from a gmt of 61 459 gene sets took about 47.5 s (Linux AMD Ryzen 4750U, 8-core, 48GB. Ubuntu 24.04.2 LTS), resulting in a matrix with >99% sparsity.

### 2.4 PLAID as a suite of other single-sample gene set enrichment scoring methods

The efficiency of PLAID stems from its use of sparse matrices. In order to improve existing methods, we have implemented functionalities to use singscore (Foroutan *et al.* 2018), GSVA (Hänzelmann *et al.* 2013), UCell (Andreatta and Carmona 2021), AUCell (Aibar *et al.* 2017), ssGSEA (Barbie *et al.* 2009), and scSE (Pont *et al.* 2019) within PLAID, using PLAID as “back-end.” The following functions have been developed within PLAID: *replaid.sing, replaid.scse, replaid.ssgsea, replaid.gsva, replaid.ucell, replaid.aucell* (Supplementary data). These functions are highly efficient in runtime and memory, generating GSESs concordant with the original methods.

## 3 Results

Single-sample gene set and pathway enrichment scoring enables the prioritization of molecular signatures of individual samples, highlighting potential targets for personalized medicine. Several methods have been developed, each with distinct strengths and limitations. The most widely used include ssGSEA (Barbie *et al.* 2009), GSVA (Hänzelmann *et al.* 2013), AUCell (Aibar *et al.* 2017), singscore (Foroutan *et al.* 2018), scSE (Pont *et al.* 2019), and UCell (Andreatta and Carmona 2021). Despite methodological variations in ranking, centering, and normalization, these methods collectively offer vast analytical flexibility (Fan *et al.* 2024). Nevertheless, except for scSE, most methods are practically infeasible in runtime and memory for large-scale data.

We address this critical issue with PLAID. Within a sample, PLAID identifies genes mapped in a gene set and calculates the GSES as the average log-intensity of genes in the gene set. PLAID does not zero-center or rank features. It leverages sparse matrices for efficient computations. We evaluated PLAID in single-cell transcriptomics [from Seurat (Hao *et al.* 2024)], bulk proteomics (Wolf *et al.* 2020), and microarray expression data (Lenz *et al.* 2008). These data span diverse scenarios in terms of signal resolution and distribution, allowing comprehensive benchmarks. We compared PLAID runtime, peak memory, and GSESs to singscore, ssGSEA, GSVA, scSE, UCell and AUCell.

In scRNA-seq data, PLAID consistently outperformed other methods at different sample and gene set sizes (Fig. 1A; Table 1, available as supplementary data at *Bioinformatics* online). PLAID scored 2864 gene sets in 1K cells in 0.17 s, which is >100× faster compared to any other method. The second-best method was scSE. Overall, the top memory-performing methods were PLAID and scSE. A similar trend was observed for 10K cells, where PLAID faster, requiring similar RAM when compared to other non-timed-out methods. When testing 61 459 gene sets in 1K cells, PLAID, singscore, UCell, and AUCell successfully completed the scoring. Other methods were timed out at 1 h. PLAID completed the run in <8 s, with <3 GB peak RAM. Singscore, UCell, and AUCell required >100× runtime compared to PLAID, with up to ∼1.5 GB additional memory. GSVA, ssGSEA and scSE were timed out and neither runtime nor peak RAM were accurately estimated (Fig. 1A; Table 1, available as supplementary data at *Bioinformatics* online). For 61 459 gene sets and 10K cells, PLAID achieved scoring within 1 h, requiring 110 s and <20 GB peak RAM (Fig. 1A; Table 1, available as supplementary data at *Bioinformatics* online). Other methods were timed out and would have required at least 5× additional computing resources.

While sparse matrices would be ideal, researchers often use dense matrices. To assess this, we profiled PLAID runtime and memory on the TCGA-BRCA dense expression data matrix (https://www.cbioportal.org, Cerami *et al.* 2012). PLAID outperformed other methods in most scenarios. It was >100× faster than AUCell and GSVA for 61 459 gene sets and 1K cells, requiring 2.4 GB, standing as an efficient alternative to other methods. Notably, scSE remains a highly memory-optimized method capable of outperforming PLAID in some cases (Fig. 1, available as supplementary data at *Bioinformatics* online). Runtime and memory usage by PLAID increase approximately linearly with the number of cells or gene sets. For 1K gene sets and 1M cells, PLAID required ∼200 s and 28 GB peak RAM (Fig. 2, available as supplementary data at *Bioinformatics* online).

PLAID single-sample GSESs are median normalized to aid cross-group comparisons (Methods). We performed comparison between PLAID’s scores and other methods. In scRNA-seq, for the first available cell, PLAID correlated well with singscore, UCell and AUCell scores (Fig. 1B). By using the average, scSE (“scse.mean”) produces GSESs highly concordant with PLAID (Fig. 1B). Lower similarities emerged with GSVA and ssGSEA; these two latter methods also exhibited low concordance with any other method (Fig. 1B). Within any single sample or cell, the raw gene set scores are not well suited for direct comparisons between samples. However, average and centered log-expression could be used. Interestingly, data centering resulted into a generalized improved concordance between PLAID and any other method (Fig. 1B). This supports the suitability of PLAID’s scores for differential testing between groups, and the possibility of cross-methods validation. Testing in a bulk proteomics dataset (Wolf *et al.* 2020), confirmed the high concordance between PLAID and scse.mean, singscore and ssGSEA (Fig. 3, available as supplementary data at *Bioinformatics* online). Low concordance emerged between PLAID and GSVA. GSVA was lowly correlated with any other methods (Fig. 3, available as supplementary data at *Bioinformatics* online) in scRNA-seq and bulk proteomics. An improved concordance emerged when centering the gene sets.

Running independent methods to validate enrichment results is a best practice. However, given the computational inefficiency of most methods, this is time prohibitive in large data. We provide a solution to this problem by equipping PLAID with functions to run popular single-sample GSES methods—*replaid.sing, replaid.scse, replaid.ssgsea, replaid.gsva, replaid.ucell, replaid.aucell.* These functions use PLAID as “back-end” to provide efficient GSES (Supplementary data: *Replicating other methods’ GSESs*). We conducted testing in scRNA-seq, proteomics and microarray data (Fig. 1C; Figs 4–9, available as supplementary data at *Bioinformatics* online). Best concordances emerged for *replaid.sing, replaid.scse, replaid.ucell* and *replaid.aucell* when compared to original methods. A relatively lower concordance, especially in scRNA-seq, is observed between *replaid.ssgsea* and *replaid.gsva* compared to original ssGSEA and GSVA (these two latter were not originally intended for single-cell data). Lower concordance is also related to the “*alpha*” and “*tau*” parameters (Figs 6–7, available as supplementary data at *Bioinformatics* online), due to approximations in rank weights and empirical cumulative distribution in PLAID. Nevertheless, we achieve good concordance in nearly all cases, with up to 10-fold gain in computational efficiency. Altogether, these data demonstrate the multi-task power of PLAID as a computationally efficient GSES method and framework for existing single-sample enrichment methods. We also provide evidence that expert R implementation that leverages optimized packages can result in improved performance.

These analyses show that PLAID is an accurate, ultrafast and memory-efficient method for single-sample GSES, producing results concordant with existing methods while being up to 100× faster and 5× more memory-efficient across datasets.

## 4 Discussions

Scoring gene sets and pathways at the single-sample or single-cell level is vital for identifying coordinated molecular changes that may drive disease. However, most of the current algorithms are not suitable for large datasets due to high memory requirements and slow run times. We introduce PLAID, an ultrafast, memory-efficient method that scores gene sets based on their average intensity. PLAID uses sparse matrices and is significantly faster compared to existing methods while maintaining highly concordant results. Uniquely, PLAID can also replicate scores from other methods, but with significantly improved efficiency. PLAID serves both as a standalone scoring tool and as a platform for running other GSES methods at substantially reduced computational demands.

GSES methods vary widely in accessibility, user-friendliness, and required expertise. While recent advances, such as GUIs and streamlined R installations, have improved usability, challenges persist in preparing and managing pathway data. PLAID addresses the critical issue of computational inefficiency, enabling large-scale gene set enrichment scoring and making functional analysis more broadly accessible. However, like most methods, PLAID does not discern activating versus inhibitory features within pathways. Single Cell Signature Scorer overcome this limitation by incorporating directional effects. ScSE also includes a memory-efficient modality (“scSE_low_mem”), able to control RAM usage at increasing number of cells.

Future work for PLAID could focus on extending its application to a broader range of omics datasets, including single-cell multi-omics profiles such as ATAC-seq and spatial transcriptomics. Integrating machine learning models with PLAID could also enhance large-scale predictive analytics for patient stratification and therapeutic target prioritization for personalized medicine approaches. Continuous optimization of algorithm’s memory management is also necessary to efficiently handle future datasets of increasing size and complexity, warranting scalability in biomedical research.

## Supplementary Material

btaf621_Supplementary_Data
